# Ligature of the Primitive Iliac Artery for Traumatic Aneurism

**Published:** 1866-05

**Authors:** Ralph N. Isham

**Affiliations:** Prof. of Operative Surgery in Chicago Medical College, etc.


*Ligature of the Primitive Iliac Artery for Traumatic Aneurism.* By RALPH N. ISHAM, M. D., Prof. of Operative Surgery in Chicago Medical College, etc.

CASE. August Tapka, Private, Co. K, 35th Wis. Vols., age 25, was admitted to the Military Department of the U. S. Marine Hospital, Sept. 30th, 1864.

He had received a bayonet wound of the right buttock through the ischiatic notch, while stationed at a camp near Milwaukee, March 18th, 1864. He bled to the extent of 16 or 20 ounces from the wound; the urine, which had to be drawn by the catheter for four days after the injury was received, contained much blood. There also occurred at once great swelling of the abdomen and right buttock. He was kept under treatment at Camp Washburn two months, when the swelling decreased and he began to walk. On the 12th of July he was reported fit for duty, and ordered to join his regiment at White River, Ark. He arrived there in a condition totally unfit for any service; the tumor rapidly returned, with much pain, in consequence of which he was hardly able to walk with the use of a cane. The swelling continuing to increase, after two days in the regimental hospital, he was sent to the general hospital at New Orleans. He states that he was then treated with applications to relieve his suffering, which he described as a "hammering pain" in the tumor with a corresponding movement in the part, which he had often observed himself. Not improving, he was furloughed, placed upon a hospital boat and sent North. A surgeon on the boat opened the tumor with a bistoury, evidently mistaking its true character; blood escaping from the wound, it was immediately closed. Oozing continued to occur from this time; the patient arrived in Chicago in an exceedingly weakened condition, and was received into this Hospital.

On admission he was suffering great pain, referred to the tumor and right leg with constitutional irritation. He was anæmic with a quick and small pulse and anxious expression of countenance, presenting, in short, the appearance of one who had suffered from great loss of blood. On inspection there was a hard, tense, red and glistening tumor occupying the right nates, extending from the crest of the ilium to the natal fold, and from the right border of the os-sacrum to and over the anterior spinous process of the ilium. The cicatrix of the original wound was nearly in its center, and beside it was the recent wound made by the surgeon who opened the tumor, dilated to the size of a half-dollar and filled with clots of blood, through which (Oct. 2d) arterial blood escaped for the first time since admission. He complains of the throbbing pain and numbness of the limb, with difficulty of voiding urine.

A bruit is heard by applying the ear over the tumor, but no pulsation is audible. On the occurrence of the first hemorrhage in the Hospital, (Oct. 2d,) an injection into the sac of a solution of perchloride of iron was resorted to, with the effect of temporarily arresting the hemorrhage. On the 5th and 6th of October, patient improving. Meanwhile, the hemorrhage again occurred, notwithstanding the repetition of the injections. He had been seen by several medical gentlemen, who at first advised palliative measures. Upon further consultation, however, it was decided to wait no longer, but that *the operation of tying the common iliac artery* be performed for his relief. Accordingly, October 7th, 2 P. M., he was placed under the influence of chloroform by Sergeant Griffith, U. S. A., Hospital Steward, and assisted by Surgeon L. H. Holden, U. S. A., Medical Director, and Dr. Terry, A. A. Surgeon, U. S. A., and others, I proceeded to perform the operation. I made a curvilinear incision from in front of the extremity of the last rib downwards and forwards to the crista ilii, then forwards above and along the crest, terminating at the anterior superior spine. The muscles were successively divided, with the fascia transversalis at the bottom of the incision, and the peritoneum being protected by two fingers introduced into the sub-peritoneal interval, the wound was enlarged to the extent of the original incision. The peritoneum was carefully lifted uninjured by the hand, together with the intestines, from the fascia iliaca, retained by an assistant, and the vessel exposed to view, not a drop of blood obscuring the parts. The ureter was lifted by the peritoneum. The vessel was ligated with a Mott's artery needle. The tightening of the ligature not only arrested the circulation in the limb, but diminished the size of the tumor so that its surface, which before was very tense, was now quite flaccid. The wound was properly closed, with the end of the ligature escaping from it, the patient placed in bed, limb in proper position, and enveloped in cotton.

Evening, 7 o'clock. Patient is resting comfortably, is free from pain, pulse 90. Bottles of warm water are kept near the limb which is enveloped in cotton, to maintain its warmth. Morphine, one-half grain. Oyster broth.

Saturday, Oct. 8th, 7 A. M. Patient slept well all night, limb warm, says he feels "very well," pulse 113. Ordered tr. verat. virid., 4 gtt. At 8 P. M., pulse 80, having passed a comfortable day. Color has returned to the limb with natural warmth. The wound is discharging pus and is in a healthy condition. Complains of some pain in stomach and bowels. Ordered a small dose of morphine, oyster broth and beef-tea.

Sunday, Oct. 9th. Is very comfortable, pulse 90, wound looking well, but an offensive discharge from sac appearing; *the clots were turned out*, and it was injected with a solution of permanganate of potassa.

Monday, Oct. 10th, 8 A. M. Pulse 80, patient feels well; all symptoms look favorable, but sanious and offensive discharges from the sac continue. Injections repeated to-day. 12. A. M. Pulse 90, limb natural, but patient is bathed in perspiration; the discharge from the sac is very offensive. 9

P. M. Is worse, pulse 115, wound of sac gangrenous, and, Tuesday, Oct. 11th, 10 A. M.,—dead.

Post-mortem examination showed a healthy condition of the artery, a well organized clot extending from ligature to aorta; no evidences of peritonitis. The wound of the artery proved to be in the anterior trunk of the internal iliac, within the sacro-ischiatic notch. The enormous sac was gangrenous. There were no appearances to account for the hæmaturia in the early history of the case. The difficulty of diagnosis in this case between gluteal aneurism and hemorrhage from within the pelvis, was only relieved by the previous history. The intelligent patient positively stated that the swelling first appeared within the crest of the ilium, where he had seen the pulsations; nor was there any additional evidence gained by a rectal examination. Whilst it was thus settled that the aneurism had its origin within the pelvis, the remaining uncertainty as to its true source decided that the operation should be on the common iliac. Had it been a gluteal aneurism, the operation to be resorted to would have been plainly that of turning out the clots and tying the artery through the sac, according to Astley Cooper's method, revived and practiced by Syme. An interesting feature in the case is, the disappearance of the tumor and its subsequent return, constituting that rare occurrence known as *secondary aneurism*, or perhaps, more strictly speaking, a recurring aneurism, for it would seem that the tumor did not occupy precisely the same position after its recurrence as before. A curious and interesting case of recurring aneurism is detailed by J. B. Cutter, M. D., of Newark, in the *American Jour. of Med. Sciences*, for Oct., 1865, after ligature of the external iliac artery, and for which the primitive was tied without success. The statistics of this operation when performed for arrest of hemorrhage or traumatic aneurism, are so very unfavorable as to cast a doubt over the mind of the judicious surgeon, as to whether it ever ought to be performed under these circumstances. In Prof. Stephen Smith's collection of cases, (*Am. Jour. of Med. Sciences*, for July, 1860,) thirty-two in number, seven recovered. Of these cases eleven were for the arrest of hemorrhage in wounds, or in surgical operations, only one of which recovered. To these should be added Dr. Cutter's, Dr. Hammond's, of San Francisco, for gluteal aneurism, and my own, making fourteen cases with one recovery. And from the history of these cases, even when a prospect of recovery is promised by the temporary disappearance of the tumor, it is quite sure to return after the anastomotic circulation is established. Thus far the operation when performed for hemorrhage or traumatic aneurism, has not offered any prospect of success, and I believe that if the ligature is admissable it must be placed at the wound by the old operation, and that the application of the ligature by Hunter's method, in those cases where the wound of the artery cannot be reached, will rarely succeed. The gangrene may be accounted for in the enormous size which the tumor attained, owing to the laxity of the parts in which it was situated, and its being subjected to much pressure and irritation during his long journey from New Orleans, producing an inflammation of the sac, and breaking up with decomposition of the clots, instead of fibrinous consolidation.

